# A comparison of smartphones to paper-based questionnaires for routine influenza sentinel surveillance, Kenya, 2011–2012

**DOI:** 10.1186/s12911-014-0107-5

**Published:** 2014-12-24

**Authors:** Henry N Njuguna, Deborah L Caselton, Geoffrey O Arunga, Gideon O Emukule, Dennis K Kinyanjui, Rosalia M Kalani, Carl Kinkade, Phillip M Muthoka, Mark A Katz, Joshua A Mott

**Affiliations:** Influenza Program, Centers for Disease Control and Prevention-Kenya, P.O. Box 606, 00621 Village Market, Nairobi, Kenya; Kenya Medical Research Institute (KEMRI), Nairobi, Kenya; Department of Disease Surveillance and Response (DDSR) Ministry of Health, Nairobi, Kenya; Center for Surveillance, Epidemiology, and Laboratory Services, Centers for Disease Control, Atlanta, Georgia USA

**Keywords:** Data collection, Electronic, Pen-and-paper, Smartphone, Quality, Cost, Timeliness

## Abstract

**Background:**

For disease surveillance, manual data collection using paper-based questionnaires can be time consuming and prone to errors. We introduced smartphone data collection to replace paper-based data collection for an influenza sentinel surveillance system in four hospitals in Kenya. We compared the quality, cost and timeliness of data collection between the smartphone data collection system and the paper-based system.

**Methods:**

Since 2006, the Kenya Ministry of Health (MoH) with technical support from the Kenya Medical Research Institute/Centers for Disease Control and Prevention (KEMRI/CDC) conducted hospital-based sentinel surveillance for influenza in Kenya. In May 2011, the MOH replaced paper-based collection with an electronic data collection system using Field Adapted Survey Toolkit (FAST) on HTC Touch Pro2 smartphones at four sentinel sites. We compared 880 paper-based questionnaires dated Jan 2010-Jun 2011 and 880 smartphone questionnaires dated May 2011-Jun 2012 from the four surveillance sites. For each site, we compared the quality, cost and timeliness of each data collection system.

**Results:**

Incomplete records were more likely seen in data collected using pen-and-paper compared to data collected using smartphones (adjusted incidence rate ratio (aIRR) 7, 95% CI: 4.4-10.3). Errors and inconsistent answers were also more likely to be seen in data collected using pen-and-paper compared to data collected using smartphones (aIRR: 25, 95% CI: 12.5-51.8). Smartphone data was uploaded into the database in a median time of 7 days while paper-based data took a median of 21 days to be entered (p < 0.01). It cost USD 1,501 (9.4%) more to establish the smartphone data collection system ($17,500) than the pen-and-paper system (USD $15,999). During two years, however, the smartphone data collection system was $3,801 (7%) less expensive to operate ($50,200) when compared to pen-and-paper system ($54,001).

**Conclusions:**

Compared to paper-based data collection, an electronic data collection system produced fewer incomplete data, fewer errors and inconsistent responses and delivered data faster. Although start-up costs were higher, the overall costs of establishing and running the electronic data collection system were lower compared to paper-based data collection system. Electronic data collection using smartphones has potential to improve timeliness, data integrity and reduce costs.

## Background

One of the goals of sentinel influenza surveillance is to minimize the impact of disease by providing useful information to public health authorities so that they may better plan appropriate prevention and control measures [1]. An effective influenza surveillance system should also be able to identify novel or emergent pathogens in the community, and promptly alert health authorities about persons most affected in order to better target programmatic responses. For surveillance to be effective, data collection systems should be able to maintain data integrity, quickly provide analysis-ready data, and be sustainable to run. In the last decade, information and communication technology has experienced immense growth and development. In the health care sector, computers and other electronic devices are being used to collect and store patient data in place of traditional pen-and-paper data collection which can involve labor-intensive data entry and limit timely analyses [2,3]. In middle-low income country surveillance contexts such as Kenya, data collected using an electronic device has the potential to produce timely and accurate data while reducing expenses on paper, storage space, and data entry time.

Using pen and paper collected data and smartphone collected data from an influenza sentinel surveillance system in Kenya, we compared the data quality, timeliness, and operating costs of the smartphone data collection system versus the pen-and-paper data collection system.

## Methods

### Sentinel surveillance system

**Figure 1:**
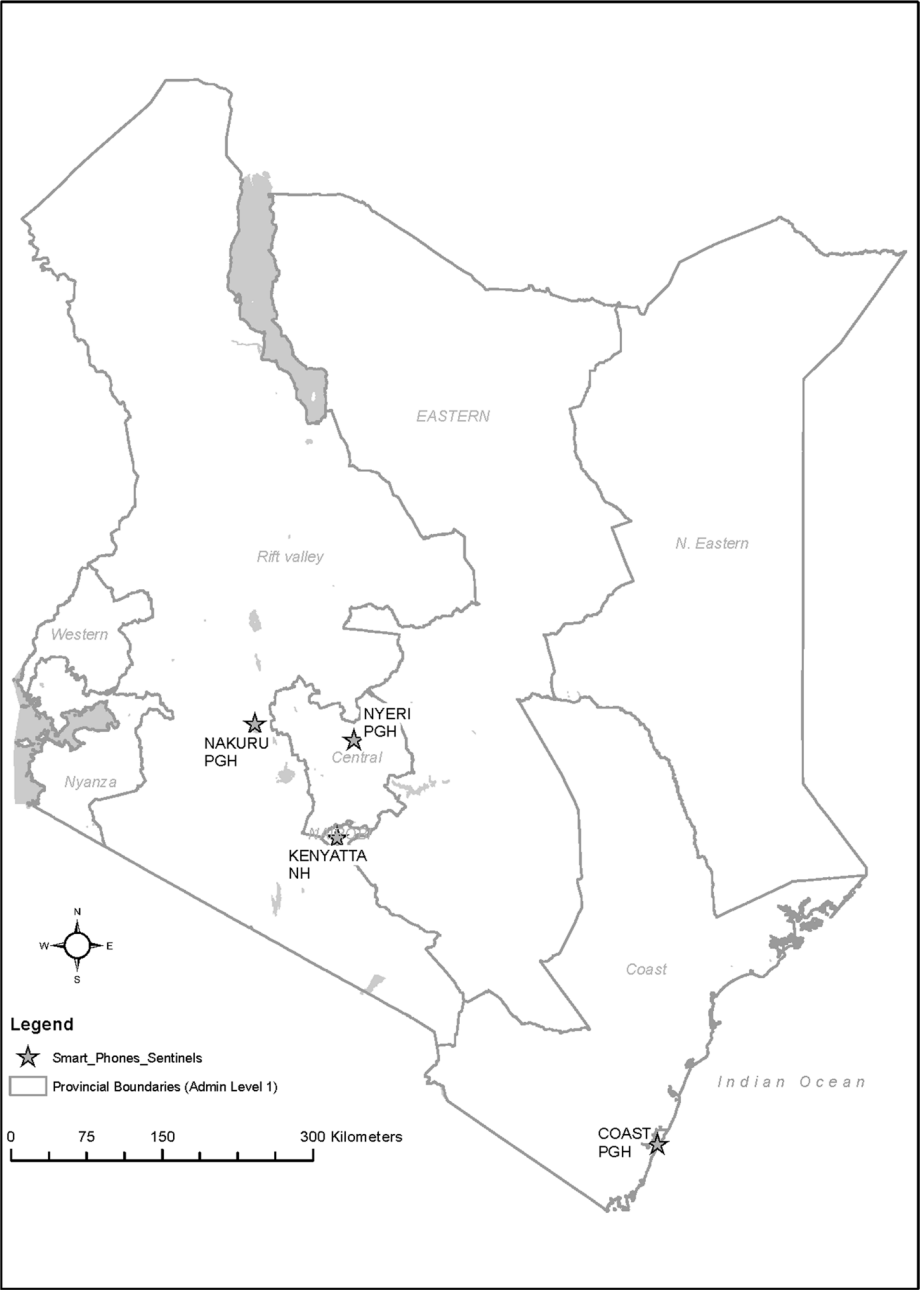
**Map showing location of influenza surveillance sentinel sites where smartphones were introduced.**

Since August 2006, the Kenya Ministry of Health (MoH), with technical support from the Kenya Medical Research Institute and Centers for Disease Control and Prevention (KEMRI/CDC), initiated influenza sentinel surveillance at 11 hospitals throughout the country. Each of these sites had a trained surveillance officer who identified inpatients meeting the World Health Organization’s (WHO’s) Severe Acute Respiratory Illness (SARI) case definition [4]. In addition, these officers also identified up to a maximum of three outpatients in the respective hospitals’ outpatient clinics per day meeting the WHO’s Influenza-like Illness (ILI) case definition. ILI surveillance stopped in July 2011 in all hospitals with the exception of KNH. In May 2011, we introduced smartphone data collection tools in four sites. These included: pediatric ward surveillance at Kenyatta National Hospital (KNH), the country’s main referral hospital located in Nairobi; and medical and pediatric ward surveillance at Coast Provincial and General Hospital (CoPGH), Nakuru PGH (NaPGH) and Nyeri PGH (NyPGH) (Figure 1). We chose these four sites to pilot the new system because of their accessibility.

#### Paper-based data collection

For every consenting patient with SARI or ILI, the surveillance officer administered a 20-question form that included closed-ended questions about patient demographics, case classification, clinical symptoms and signs, epidemiological risk factors (including recent contact with sick or dead persons with respiratory illness, smoking, and occupational exposure), and vital signs on admission. Respiratory specimens were then collected for laboratory testing. Each questionnaire was assigned a unique identification number that was linkable to the respiratory virus laboratory test results. The surveillance officers underwent annual protocol training. The MoH with support from KEMRI/CDC also provided supervision visits at each sentinel site on a quarterly basis.

For every consenting case, the surveillance officer assigned each patient a unique identification number and completed the paper questionnaire. These questionnaires were then packaged and sent to a central data office in Nairobi on a weekly basis. In the data office, data clerks entered the data manually into a Microsoft Access database that had data entry checks in place. A data analyst then went through the data and performed systematic quality checks by running scripts to flag errors and inconsistencies which were then reconciled by verification with the hard copy forms. Depending on the workload, questionnaires were at times batched before being entered into the database.

#### Electronic data collection

We used the Field Adapted Survey Tool (FAST) kit developed by GeoAge Inc (Jacksonville, FL) to program all the surveillance questions into the HTC Touch Pro2 smartphones with an internal memory of 288 MB RAM, 512 MB ROM. At the end of every workday, surveillance officers sent the data by internet, using an internal 3G smartphone connection, to a secure central server. During May-June, 2011, sites switched from paper-based to smartphone data collection. Before the switch occurred, surveillance officers underwent two-day training on the new system. We provided on-site supervision for up to one week to ensure a smooth transition from pen-and-paper-based to smartphone-based data collection.

In this new system, consenting patients were assigned a unique identification number and data were collected through entry into a smartphone using the FAST software with touch screen and keyboard features. Administrative, demographic and epidemiological variables (e.g. unique identification number, case classification, patient’s age and pregnancy status (for female respondents)) included programmed checks and restrictions to assure data quality. Some of the programmed checks required the surveillance officer to answer these questions in order to move forward in the survey. In addition, range-value restrictions were established to prevent out-of-range entries for date of data entry, age, temperature, oxygen saturation and weight. If an unacceptable response was entered, (for example, a response inconsistent with a previous entry) an error message would appear and the officer had to recheck the response and correct the inconsistency before continuing the survey. Once data was uploaded into the server, the data analyst performed quality assurance procedures to evaluate data completeness and duplication of unique identification numbers.

### Record selection

Using computer generated random numbers, we identified smartphone records collected between May 2011 and June 2012 at the four sentinel surveillance sites. We first identified the site with the least smartphone-based records available during that time period (Nyeri PGH, which had 220 records), and then randomly identified 220 smartphone-based records from each of the other three sites. We then randomly selected 220 original pen-and-paper records collected at each of the four sites during the time period of January 2010 to June 2011. In order to compare the two systems, we evaluated quality and timeliness of data entry. We also estimated the costs of running each of the data collection systems.

### Data analyses

#### Data quality

We evaluated the completeness and the percentage of erroneous and inconsistent responses in the questionnaires. For pen-and-paper records, we used the original responses recorded by the surveillance officers prior to data entry and cleaning. For each question, we identified and counted missing answers, inconsistent responses and also identified out of range values in temperature and respiratory rate measurements. Temperature values greater than 43°C (110 F) and respiratory rate counts greater than 160 breaths per minute, respectively, were considered erroneous.

In this analysis we classified the questions into the following thematic areas: case classification, clinical symptoms, past medical history, exposure factors and vital signs. We counted missing answers to all the questions in each of these areas. For each thematic area, the total count of missing answers per question (for non-mandatory questions) were summed and considered as the numerator. The summation of all questions in the thematic area was considered as the denominator. In the pen-and-paper data set, we separately determined the completeness of those questions that required a mandatory answer on the smartphone application (date of interview, case classification, sex and pregnancy status if the respondent was female). We used Poisson regression analysis to compare incidence rate ratios (IRR) of the occurrence of missing answers and errors in the smartphone and pen-and-paper datasets, respectively. We adjusted the IRR for case classification (SARI vs. ILI) and sentinel site location as potential confounders. A p-value ≤0.05 was considered significant in all the statistical analyses.

#### Timeliness of data

We compared median time from data collection to data entry into central database for the two systems. This was done by determining the date when data was collected and subtracting this from the date when data was entered (for pen-and-paper method) or uploaded into the data base (for smartphone method). The smartphone data collection system initially was not programmed to record the date when data were uploaded; this was however put in place in July 2013. We therefore only used smartphone records collected between July 2013 and September 2013 to assess timeliness of data between the two systems. Wilcoxon signed-rank tests were used to assess the difference between the two medians.

#### Costs of establishing and running paper based and smartphone data collection systems

We estimated the cost of starting-up and operating each of the data collection systems based on costs established by the KEMRI procurement system and payroll. Costs were categorized into start-up costs (if they were only incurred once, like the cost of equipment) and running costs (if they were recurrent, like the costs of paper and phone connection fees). Once the start-up and running costs for the paper-based and electronic systems were determined, we estimated the total costs of setting up and operating each system for a period of one and two years, assuming a stable number of annual cases detected based on the average of the number of cases detected in 2010 and 2011. We also assumed that should the smartphone got broken or stolen, the cost of replacing it would be covered by insurance cover.

#### Ethical considerations

The Kenya Ministry of Health (KMoH) issued a document stating that sentinel surveillance for influenza, including follow-up in-hospital surveillance, should be considered part of routine public health surveillance, and therefore did not require formal ethical review. Because the activity was classified as routine surveillance, the KMoH considered verbal consent to be adequate. Verbal consent was obtained from all patients before questionnaires were administered and specimens were collected. For children, verbal consent was obtained from guardians. The authors did not participate in data or specimen collection. Data was anonymized upon collection, and authors did not have access to identifying information.

## Results

We analyzed 1,760 surveillance questionnaires, including 880 pen-and-paper questionnaires and 880 smartphone questionnaires. The majority of questionnaires (89%) were from cases aged 2–59 months. Of the 1,760 questionnaires, 413 (24%) met the ILI case definition and 1,344(76%) met the SARI case definition while 3 questionnaires did not have the case classification specified (Table 1). There was no difference in the distribution of ILI cases by age between data collected using smartphones and pen-and-paper (p = 0.16). There was also no difference in distribution of SARI cases by age across data collection methods (p = 0.15). There were more ILI cases recorded using pen-and-paper than smartphones.

**Table 1:** **Characteristics of influenza sentinel surveillance data collected using smartphones and pen-and-paper, 4 sites, Kenya, 2011-2012**

**Characteristic**	**Total (%** ^**~**^ **)**	**Smartphone data (%** ^**^**^ **)**	**Pen-and-paper data (%** ^**^**^ **)**
**Gender**			
Male	975 (55)	501 (51)	474 (49)
Female	783 (45)	379 (48)	404 (52)
*Total* ^*#*^	*1758*	*880*	*878*
**Age of ILI and SARI Cases**			
Age 0-2mo	69 (4)	36 (52)	33 (48)
Age 2mo-5 yrs	1564 (89)	775 (44)	789 (56)
Age ≥5 yrs	127 (7)	69 (54)	58 (46)
*Total*	*1760*	*880*	*880*
**Age of ILI Cases**			
Age 0-2mo	10 (2)	4 (40)	6 (60)
Age 2mo-5 yrs	371 (90)	63 (17)	308 (83)
Age ≥5 yrs	32 (8)	5 (16)	27 (84)
*Total meeting ILI case definition* ^*$*^	*413*	*72 (17)*	*341 (83)*
**Age of SARI cases**			
Age 0-2mo	59 (4)	32 (54)	27 (46)
Age 2mo-5 yrs	1192 (89)	712 (60)	480 (40)
Age ≥5 yrs	93 (7)	64 (69)	29 (31)
*Total meeting SARI case definition* ^*$*^	*1344*	*808 (60)*	*536 (40)*
**ILI cases by site** ^$*^			
KNH	103 (25)	43 (42)	60 (58)
Coast PGH	76 (18)	7 (9)	69 (91)
Nakuru PGH	147 (36)	17 (36)	130 (88)
Nyeri PFH	87 (21)	5 (6)	82 (94)
*Total meeting ILI case definition* ^*$*^	*413*	*72*	*341*
**SARI cases by site** ^*^			
KNH	337 (25)	177 (53)	160 (47)
Coast PGH	364 (27)	213 (59)	151 (41)
Nakuru PGH	292 (22)	203 (70)	89 (30)
Nyeri PFH	351 (26)	215 (61)	136 (39)
*Total meeting SARI case definition* ^*$*^	*1344*	*808*	*536*

*Significant differences (Fisher’s exact test).

^#^Two cases missing gender.

^$^Three cases missing case classification.

^~^Column %.

^^^Row %.

### Completeness and logical consistency of data

For questions without mandatory data entry requirements as per the smartphone’s operating software programing, overall, there was a higher incidence of incomplete records in the pen-and-paper data (1%) than in the smartphone data (0.1%) (adjusted incidence rate ratio (aIRR): 7, 95% CI: 4.4-10.3) . There was also a higher incidence of errors and inconsistent answers in data collected using pen and paper 8.3% than that collected using smartphones 0.4% (aIRR: 25, 95% CI:12.5-51.8). For the questions with mandatory data entry requirements as per the smartphone system’s programing, there were 10 (0.4%) records in pen-and-paper records with missing answers (Table 2).

**Table 2:** **Completeness and logical consistency of data records collected using smartphone and pen-and-paper data collection systems, Kenya, 2011-2012**

	**Smart phone**		**Pen and paper**				
**Question type**	**Total number of questions assessed**	**Number of errors/incomplete/inconsistent records (%)**	**Total number of questions assessed**	**Number of errors/incomplete/inconsistent records (%)**	Adjusted IRR*	**p value**	**95% CI**
*Questions requiring responses without programmed checks on the smartphone version*							
Incomplete records							
Questions on clinical symptoms	11,440	11 (0.1)	11,440	54 (0.5)	4.2	<0.01	2.1-8.3
Questions assessing past medical history	1,760	6 (0.3)	1,760	5 (0.3)	0.9	0.82	0.3-2.9
Questions on exposure history	3,520	6 (0.2)	3,520	108 (3.1)	17.4	<0.01	7.6-39.8
Questions on vital signs	1,760	2 (0.1)	1,760	14 (0.8)	5.6	0.03	1.2-26.3
**Total**	**18,480**	**25 (0.1)**	**18,480**	**181 (1.0)**	**7**	**<0.01**	**4.4-10.3**
Errors and inconsistent answers							
Questions assessing past medical history	243	1 (0.4)	227	178 (78.4)	200	<0.01	28.0-1431.2
Questions on exposure history	6	2 (33.3)	6	3 (50.0)	1	0.86	0.2-8.2
Questions on vital signs	1,760	5 (0.3)	1,760	5 (0.3)	1	0.48	0.2-2.4
**Total**	**2,009**	**8 (0.4)**	**1,993**	**186 (8.3)**	**25**	**<0.01**	**12.5-51.8**
*Questions requiring responses with programmed checks on the smartphone version*							
Date of interview	-	-	880	0 (0)	-	-	-
Case classification	-	-	880	2 (0.2)	-	-	-
Gender	-	-	880	2 (0.2)	-	-	-
Pregnant if female	-	-	9	6 (66.7)	-	-	-
**Total**	-	-	**2,649**	**10 (0.4)**	-	-	-

### Timeliness of data

Of the 500 smartphone and 880 pen-and-paper records with data on the date of collection and upload/entry available, smartphone-collected data were uploaded from the sentinel sites into the central database in Nairobi at a median duration of 7 days (range 1–13 days) following data collection. It took a median duration of 21 days (range 4–56 days) to have pen-and-paper records entered into the central database (p < 0.01).

### Estimated costs of running the two systems of data collection

**Figure 2:**
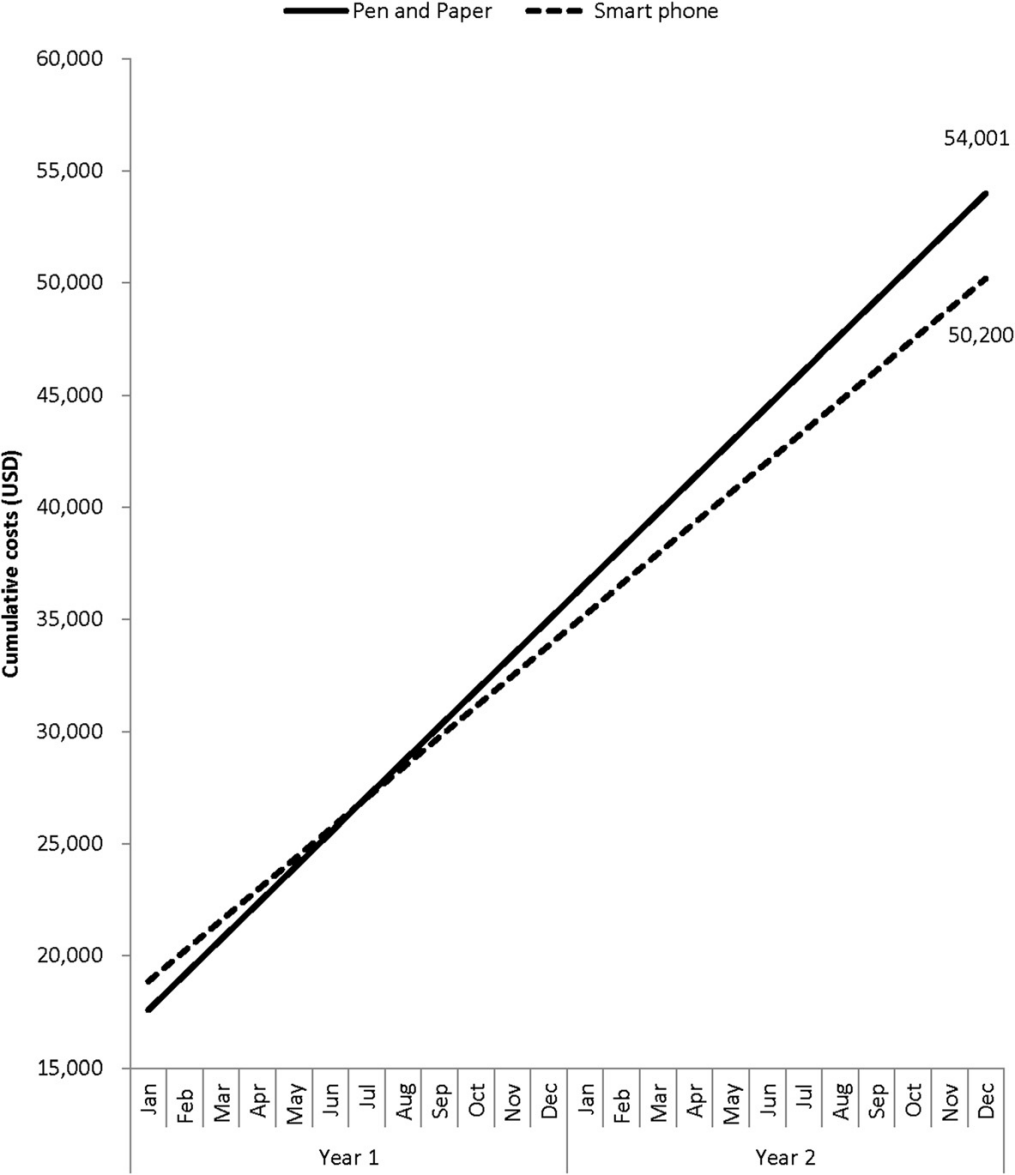
**Projected costs of running a smartphone and paper based data collection system, Kenya, 2011–2012.**

The estimated start-up cost for the smartphone data collection system was higher (USD $17,500) than that of pen-and-paper data collection system (USD $15,999) (Table 3). However, combined start-up and running costs for the smartphone system were less than that of pen-and-paper system ($ 16,350 vs. $ 19,001 respectively) in year 1. This difference in total operating costs widened as the costs were estimated over a two year period ($32,700 vs. $38,002). Over the two year period, the smartphone system was $3,801 (7.0%) more economical to establish and run compared to pen-and-paper system. When considering the start-up costs incurred, the smartphone system became less expensive to operate than the pen-and-paper data collection system at the 7^th^ month of the first year (Figure 2).

**Table 3:** **Estimated start-up and running costs of establishing and operating a smartphone and paper-based data collection system for one year and for two cumulative years**

	**Pen and paper data collection system**					**Smart-phone data collection system**					**Differences between the two systems**		
	**Unit cost (Kshs)**	**Quantity needed per year**	**Total cost year 1 (USD)**	**Total cost year 2 (USD)**	**Cumulative total costs year 1 and 2 (USD)**	**Unit cost (Kshs)**	**Quantity needed per year**	**Total cost year 1 (USD)**	**Total cost year 2 (USD)**	**Cumulative total costs year 1 and 2 (USD)**	**year 1**	**year 2**	**Cumulative**
Fixed costs													
Computer (one computer)	1,385	3	4,155	-	4,155	1,385	1	1,385	-	1,385			
Scanner (one scanner)	900	1	900	-	900	-	-	-	-	-			
Server	10,125	1	10,125	-	10,125	10,125	1	10,125	-	10,125			
Filing cabinets (one)	169	1	169	-	169	-	-	-	-	-			
Printer (one)	650	1	650	-	650	-	-	-	-	-			
FAST software	-	-	-	-	-	5,000	1	5,000	-	5,000			
HTC smart-phones	-	-	-	-	-	220	4	879	-	879			
Extended life battery	-	-	-	-	-	10	4	40	-	40			
Data sim cards	-	-	-	-	-	1	4	5	-	5			
Insurance (smart phones)	-	-	-	-	-	17	4	66	-	66			
*Total fixed costs*			*15,999*		*15,999*			*17,500*		*17,500*	*(1,501)*	*-*	*(1,501)*
Running costs													
Printing paper (one paper)	0	3,662	46	46	92	-	-	-	-	-			
Printing cost (one page)	0	7,324	275	275	549	-	-	-	-	-			
Data entry (clerk) labor cost (one questionnaire)	1	3,662	2,335	2,335	4,669	-	-	-	-	-			
Data entry and cleaning costs	0	3,662	595	595	1,190	-	-	-	-	-			
Data analyst (one year contract)	15,750	1	15,750	15,750	31,500	15,750	1	15,750	15,750	31,500			
Ball pen ( one pen)	0	6	1	1	2	-	-	-	-	-			
Air time (per month per site)	-	-	-	-	-	13	48	600	600	1,200			
*Total running costs*			*19,001*	*19,001*	*38,002*			*16,350*	*16,350*	*32,700*	*2,651*	*2,651*	*5,302*
**Total fixed costs plus running costs**			**35,000**	**19,001**	**54,001**			**33,850**	**16,350**	**50,200**	**1,150**	**2,651**	**3,801**

## Discussion

In disease surveillance, data collection systems should be sustainable, easy to use and able to provide timely data and consistently maintain data integrity [5-7]. Our study demonstrated that a smartphone data collection system using FAST outperformed pen-and-paper systems across each of these domains for influenza sentinel surveillance in Kenya.

Data collected using smartphones were more likely to be complete and had fewer inconsistencies and errors compared to pen-and-paper data. These findings may be attributed to the availability of programmed quality checks for select questions in FAST program and may not be specific to a particular smart phone platform per se. A study from Fiji that evaluated public surveillance data collected electronically using hand-held personal digital assistants (PDAs) found similar results on data quality and completeness [8]. In this Fiji study, data quality was measured using error rates (logical range errors/inconsistencies, skip errors, missing values, date or time field errors and incorrect data type) as collected using pen-and-paper versus PDA. Similar to this study, electronically collected data in the Fiji study were more complete and had fewer errors than paper collected data [8]. The ability to include quality checks on a data entry in electronic data collection system makes it more versatile compared to pen-and-paper data collection system [3]. In addition, errors were further minimized by directly uploading electronic data into the database without going through the process of secondary data entry, which can potentially introduce additional errors [9,10].

Studies have shown that electronically collected data take less time to become available for analysis compared to pen-and-paper collected data [3,11]. However, in our study, the time taken to have electronically collected data available in the database could have further been shortened. Poor network coverage in certain areas within the health facilities necessitated that data be saved in the smartphone’s memory and later uploaded into the server at convenient places where there was good network coverage. Occasional server communication breakdowns may have also increased the time taken for this data to be uploaded into the database. Despite these obstacles, our electronic data collection systems still reduced the time needed for routine data to be available for analysis by two weeks.

The cost of establishing and running the electronic data collection system was initially higher than that of paper-based systems. This was largely because of the higher cost of electronic equipment and operating software. Similar findings have been observed in a study conducted by Thriemer *et al.* where capital costs of setting up an electronic data collection system were higher than that of establishing paper-based system [12]. However, the overall costs of running the electronic data collection system were indeed lower and became more economical than paper systems by the 7^th^ month of surveillance in Kenya. This can be explained by the elimination of the need to have secondary data entry and intensive data cleaning as has been explained in other studies [12,13]. Since disease surveillance platforms are likely to be ongoing by definition, programs may consider using electronic data collection systems that are more sustainable.

Although not formally evaluated in our study, the surveillance officers reported that use of smartphones to collect data was faster, easier to follow and more convenient as they did not have to carry the weight of paper based questionnaires. They also reported need for less space to store their data collection tools.

### Limitations

Our study had several limitations. The two systems of data collection were compared using data collected separately from different patients at different time periods. One may argue that the surveillance officers may have improved on their data collection skills over time and hence fewer errors/inconsistent responses by the time data were collected using smartphones. However, this analysis was done at a time when the sentinel surveillance sites had been running for over 4 years by the same surveillance officers. By the time the study was done, the surveillance officers’ skills in data collection had been optimized and therefore unlikely to impact on this comparison.

While electronic data collection systems may have data quality checks, it is possible that the interviewers may have keyed in responses that are not consistent with those given by the interviewee. This would compromise data quality and could not be measured in this case, but would also be more related to the quality of data collection personnel rather than data collection platform.

The costs of establishing and running the two data collection systems were based on rates provided by KEMRI procurement and payroll system. It is possible that equipment costs and personnel rates may vary from region to region thus affecting generalizability of our findings in this regard.

Due to the limited memory available in our smartphones, data was deleted from the phone’s memory once it was uploaded into the central server. It was therefore impossible to track the unique identification numbers of enrolled cases as the application was not designed to assign unique patient identity numbers automatically. This shortcoming can be overcome by use of nascent technologies such as cloud computing, where virtual servers and computing power from existing providers can be utilized to store and retrieve data when needed [14]. In our study the surveillance officers maintained a log of all patients enrolled in a patient register, where unique identification numbers were assigned before entering it into the smartphone at enrollment. This was to avoid keying in wrong patient identification numbers during follow-up data collection.

The FAST software used in our study was also limited in its ability to accommodate complex programs required for larger research studies. The numbers of branching options needed when designing a logical flow of questions are limited. This makes it difficult to display select questions for specific subpopulations. For example questions directed to women in the reproductive age group were displayed for all female patients regardless of age. This would suggest that smartphone based systems operated using FAST software may be more suitable for simple routine surveillance systems than for larger research platforms.

Lastly, the sustainability of electronic surveillance systems may be in part dependent on current availability of software programs. In this case the production of FAST software has since been terminated and there are no new software upgrades. Other smartphone application software will now need to be evaluated to determine if they are suitable to collect disease surveillance data. Notwithstanding, many of the principles of improved data quality and cost effectiveness that were demonstrated by FAST loaded on HTC Smartphones in this case would apply to other software programs and smart phone brands.

## Conclusions

Despite these challenges, our study demonstrates that electronic data collection using smartphones can be effectively implemented in routine influenza sentinel surveillance in a tropical and developing setting. In this setting a smartphone-based system provided the end users with more timely, cost-effective, and higher quality data.

## Abbreviations

aIRR: Adjusted incidence rate ratio
CDC-Kenya: Centers for Disease Control and Prevention-Kenya
CoPGH: Coast Provincial and General Hospital
DDSR: Department of Disease Surveillance and Response
FAST: Field adapted survey tool kit
ILI: Influenza like illness
KEMRI: Kenya Medical Research Institute
KNH: Kenyatta National Hospital
MoH: Ministry of Health
NaPGH: Nakuru Provincial and General Hospital
NyPGH: Nyeri Provincial and General Hospital
SARI: Severe acute respiratory illness
WHO: World Health Organization
